# Visual Evoked Cortical Potential (VECP) Elicited by Sinusoidal Gratings Controlled by Pseudo-Random Stimulation

**DOI:** 10.1371/journal.pone.0070207

**Published:** 2013-08-05

**Authors:** Carolina S. Araújo, Givago S. Souza, Bruno D. Gomes, Luiz Carlos L. Silveira

**Affiliations:** 1 Instituto de Ciências Biológicas, Universidade Federal do Pará, Belém, Pará, Brazil; 2 Núcleo de Medicina Tropical, Universidade Federal do Pará, Belém, Pará, Brazil; Tokai University, Japan

## Abstract

The contributions of contrast detection mechanisms to the visual cortical evoked potential (VECP) have been investigated studying the contrast-response and spatial frequency-response functions. Previously, the use of m-sequences for stimulus control has been almost restricted to multifocal electrophysiology stimulation and, in some aspects, it substantially differs from conventional VECPs. Single stimulation with spatial contrast temporally controlled by m-sequences has not been extensively tested or compared to multifocal techniques. Our purpose was to evaluate the influence of spatial frequency and contrast of sinusoidal gratings on the VECP elicited by pseudo-random stimulation. Nine normal subjects were stimulated by achromatic sinusoidal gratings driven by pseudo random binary m-sequence at seven spatial frequencies (0.4–10 cpd) and three stimulus sizes (4°, 8°, and 16° of visual angle). At 8° subtence, six contrast levels were used (3.12–99%). The first order kernel (K1) did not provide a consistent measurable signal across spatial frequencies and contrasts that were tested–signal was very small or absent–while the second order kernel first (K2.1) and second (K2.2) slices exhibited reliable responses for the stimulus range. The main differences between results obtained with the K2.1 and K2.2 were in the contrast gain as measured in the amplitude versus contrast and amplitude versus spatial frequency functions. The results indicated that K2.1 was dominated by M-pathway, but for some stimulus condition some P-pathway contribution could be found, while the second slice reflected the P-pathway contribution. The present work extended previous findings of the visual pathways contribution to VECP elicited by pseudorandom stimulation for a wider range of spatial frequencies.

## Introduction

It is suggested that the generation of visual evoked cortical potentials (VECP) is the result of activation of different parallel visual pathways, such as the M- (magnocellular) and P- (parvocellular) pathways, which have different retinal origins, project to different layers of the lateral geniculate nucleus, and then to different compartments of the primary visual cortex [1]–[9]. The neurons of these visual pathways have receptive fields of different sizes, sample the visual field with different sampling densities, and respond differently to several parameters of visual stimuli such as spatial and temporal contrast as well as achromatic and chromatic content [10]. It is then assumed that by carefully manipulating visual stimulation and studying VECP amplitude as a function of certain stimulus parameters such as spatial contrast and spatial frequency will provide important clues about the contribution of the M and P pathways to the evoked response.

VECP contrast-response functions that saturate at high contrasts have been associated to the M-pathway activation [3], [4], [11] due to the similarity to the functions obtained from single recordings of M cells in the primate retina and lateral geniculate nucleus [12], [13]. Double-slope functions were described as activation of two contrast detection mechanisms [1], [2], [4], [6], [14]–[16]. It has been hypothesized that the slope at high contrast represents the combined activity of the P- and M-pathway, whilst the slope at low contrast solely represents the M-pathway activity [1], [2], [4], [6], [14]–[16].

In the beginning of 1990 decade, a new approach was applied to the VECP studies called multifocal electrophysiology [17], [18]. In this approach, the periodic stimulation used in the conventional way was substituted by an pseudo-random stimulation and the VECP was extracted by cross-correlation between the electroencephalographic recordings and the pseudo-random sequence used for the stimulation. The cross-correlated responses were called kernels.

After the development of multifocal electrophysiology, the use of pseudo-random binary sequences to control the stimulation and to obtain cross-correlating responses with this sequence has been applied to investigate the visual pathways role in the VECP generation. Fortune & Hood [19] stated that conventional VECP cannot be simply related to the VECP elicited by pseudorandom stimulation due to possible differences of cortical sources.

Klistorner et al. [11] used binary m-sequences to provide temporal luminance modulation of a central field stimulus to elicit VECP. They found that the first order kernel had large amplitude in low and high contrasts whilst at intermediate contrast levels the VECP had small amplitude. They suggested the existence of two mechanisms that cancel each other at intermediate contrasts and that each one dominated the VECP at low or high contrast, respectively. They also found that the amplitude of the K2.1 saturated at high contrast, while the amplitude of the K2.2 linearly decreased as a function of contrast. They suggested that the activity of the M- and P-pathway differentially dominated the cortical response in such a way to generate these two kinds of VECP signatures. Baseler & Sutter [18] studied the VECP generated by pattern reversal stimuli, temporally modulated by an m-sequence, and spatially distributed as a dartboard pattern. They extracted two VECP components that potentially represent the M- and P-pathway activity due the shapes of their contrast response functions. In addition, they found that the amplitude ratio between the P and M components was high at the center of the visual field and decreased towards visual field periphery. Other studies obtained contrast-response functions well fitted by hyperbolic functions using dartboards to elicit multifocal VECP [19]–[23]. Baseler and Sutter [18] and Hood et al. [22] varied the number of checks per patch in order to investigate the spatial frequency influence on the multifocal VECP, but there were few changes in the cortical response.

It should be noted that in Klistorner et al. [11] only the temporal change of luminance contrast was studied, and the investigation of spatial properties influence in the kernels used dartboard stimuli with pseudo random contrast reversal as used in Baseler & Sutter [18], which turns difficult the characterization of the contribution of different spatial frequencies to the VECP elicited by pseudo-random stimulation. Classical stimuli, as sinusoidal gratings, could be used to facilitate the investigation of the spatial frequency influences on the cortical response elicited by pseudo-random stimulation.

Momose [24] used a single grating stimulation modulated by an m-sequence to elicit VECP. The implicit time of VECP binary kernels was correlated to steady-state VECP amplitude elicited by checkerboard stimulus from 0.5 to 4 cpd at 4–32 Hz. This study concluded that the first slice of the second order binary kernel (K2.1) was more similar to the VECP data obtained from 32 Hz, reflecting M-pathway activity, while the second slice of the second order kernel (K2.2) was more similar to the VECP obtained at 4 and 16 Hz indicating a possible interaction of M- and P-pathway activities.

The present work studied VECPs elicited by single pattern stimulation temporally modulated by pseudorandom binary sequences across the spatial frequency and contrast domains. The use of sinusoidal gratings permits to extend the investigation of the spatial frequency influence in the VECP elicited by pseudo-random stimulation. Abstracts of the present work were previously published in scientific meetings annals [25], [26].

## Materials and Methods

### Subjects

Nine subjects (23.3±2.5 years old) with normal or corrected visual acuity to 20/20 were binocularly tested. None of the subjects had previous visual or neurological diseases. All subjects were verbally informed about the study and invited to participate. All of them gave written consent to participate in this study. This research was performed following the Brazilian and international regulations regarding ethics in research with human subjects. It was reviewed and approved by the Committee for Ethics in Research from Núcleo de Medicina Tropical of Universidade Federal do Pará (Protocol # 023/2011).

### Stimulation

We used achromatic horizontal sinusoidal gratings subtending 4°, 8°, and 16° of visual angle in a square field at seven spatial frequencies, from 0.4 to 10 cpd. For 8° visual angle stimuli were also presented at six contrast levels, from 3.125% to 99% Michelson contrast. The grating was centred on fixation.

All stimuli were presented in a CRT display (Barco, 75 Hz, 1280×1204 pixels). Stimulus mean chromaticity and mean luminance were the same as the background chromaticity and luminance (CIE1931 chromaticity: x = 0.31, y = 0.31; mean luminance: 40 cd/m^2^). They were measured with a CS-100A Colorimeter (Minolta, Osaka, Japan). For both experiments, a binary m-sequence (2^14^-1 elements) controlled stimulus temporal presentation. The m-sequence states 1 and 0 showed the grating stimulus differing in 180° phase reversal.

### Recording

The recording was performed using one channel of gold surface electrodes placed in Oz (active electrode), Fz (reference electrode), and Fpz (ground electrode) [27]. The signals were amplified x50,000 and on line filtered between 0.1–100 Hz. Commercially available equipment Veris Science 6.10 (ElectroDiagnostic Imaging – EDI, Redwood City, CA) was used for stimulation, recording, and data extraction. The software performed a cross-correlation technique between the electroencephalographic recording and a sequence derived from m-sequence in order to obtain the kernels elements [28]. The first order kernels are linear impulse responses while the second order kernels represent interaction between responses in specific pairs of stimulation intervals (see details about the kernel interpretation and extraction in Sutter, 2001). We extracted three kernel series: the first order kernel (K1), the second order kernel first slice (K2.1), and the second order kernel second slice (K2.2). After the extraction, the waveforms were low-pass filtered at 50 Hz.

In order to decrease the inter-subject variance of the data, for each subject at all stimulus conditions, we divided all the amplitude values of the recordings by the maximum value found among all the recordings of a subject. In order to investigate the spatial frequency and contrast influences we evaluated kernel RMS amplitude in the time period from 70 to 170 ms as indicated by Equation 1, because most of the VECP signal was included in this period. As we observed that K2.1 had less inter-subject variability and different components across the spatial frequency domain, we measured the peak-to-baseline amplitude and implicit time to peak of the VECP components for all stimulus conditions
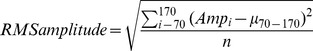
(1)


Where *Amp* is amplitude in the interval between 70 and 170 ms, µ is the average of the amplitude in the interval between 70 and 170 ms, and *n* is number of points in the analyzed interval.

### Principal Component Analysis

We performed a principal component analysis in the subjects waveforms obtained for each stimulus condition. This analysis was done with MATLAB (Mathworks, Natick, MA) using *svd()* function that computes the matrix singular value decomposition [29]. We studied how the first and second principal component contributes to the VECP in the different contrast and spatial frequency combinations.

### Contrast Response Analysis

Initially, we fitted the contrast response functions of K2.1 and K2.2 as well as K2.1 and K2.2 first and second principal components by power functions
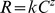



Where R is the response, C is the contrast, k is a scaling factor, and z is the exponent. In this analysis, we compared the function z values to have an estimate of amplitude saturation when contrast was raised.

Then, we fitted the same data with Michaelis-Menten functions
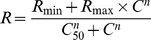



Where R is the response, R_min_ and R_max_ are the minimum and maximum responses, C is contrast, and C_50_ is contrast at half amplitude. In order to estimate the contrast gain we used
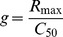



Where g is the contrast gain, R_max_ and C_50_ are as defined above.

## Results

### Spatial Frequency and Contrast Influence on the RMS Amplitude of VECP Kernels

**Figure 1:**
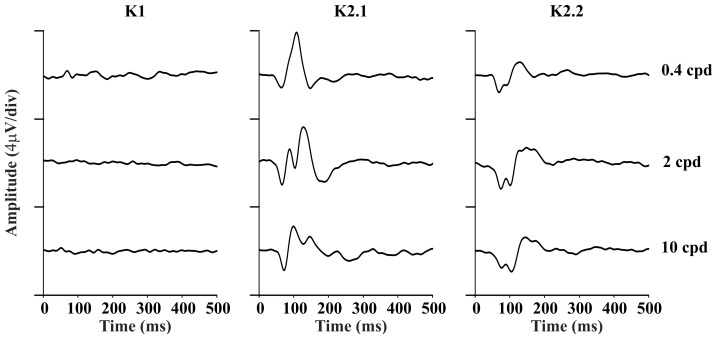
Mean VECP kernel waveforms obtained from 9 subjects at three spatial frequencies. Left column: first order kernels (K1). Center column: second order kernel first slices (K2.1). Right column: second order kernel second slices (K2.2). Due to their amplitude versus spatial frequency (Fig. 2) and amplitude versus contrast (Figure 3) functions we have suggested that K2.1 is mainly dominated by the M-pathway response or a mixture of M- and P-pathway influence, whereas K2.2 is mainly dominated by the P-pathway response (see the text for more details).

The mean waveforms of the K1, K2.1, and K2.2 are shown in Figure 1 at three spatial frequencies and high contrast (99%). K1 had very small amplitude or was entirely absent across the spatial frequency domain, while the waveforms of the K2.1 and K2.2 were robust and measurable.

**Figure 2:**
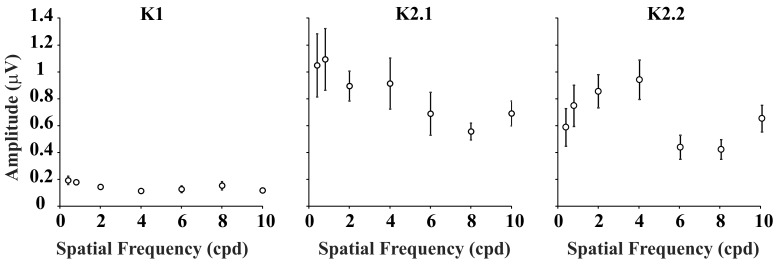
Mean RMS amplitude of VECP kernels across the spatial frequency domain at high contrast stimulation. First order kernels showed very small or no signal at all stimulus conditions (K1, left). The highest response of the second order kernel first slice occurred at low spatial frequencies (K2.1, center). The highest response of the second order kernel second slice occurred at intermediate spatial frequencies (K2.2, right). Error bars are standard errors of the means (SEM).

The mean RMS amplitude in the time period expected for the cortical responses for different kernels as a function of the spatial frequency at high contrast are shown in the Figure 2. The K1 had low mean amplitude at all spatial frequencies. The K2.1 had the highest mean amplitude at lower spatial frequencies. The K2.2 had highest mean amplitude at intermediate spatial frequencies which decreased at low and high spatial frequencies.

**Figure 3:**
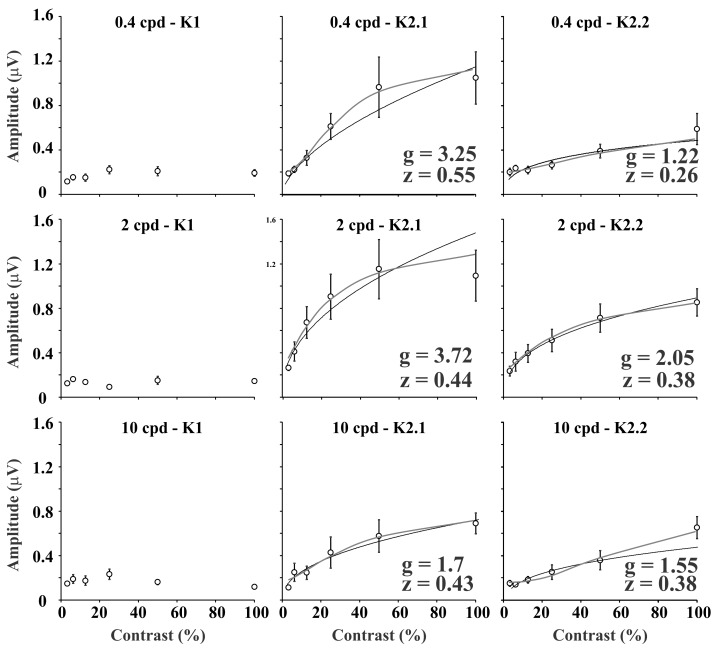
Mean RMS amplitude of VECP kernels for different contrast at three spatial frequencies (0.4, 2, and 10 cpd). First order kernels showed small or no signal at all stimulus conditions (K1, left). Second order kernel first slice (K2.1, center) and second slice (K2.2, right) amplitude saturated at high contrast at all spatial frequencies, the effect being more robust at low and intermediate spatial frequencies. We have fitted the contrast response mean values with power functions (black curves) and estimated their saturation index (z). K2.1 functions have larger z than K2.2 functions, especially at low spatial frequency. To exploit further this issue, we fitted the data point with Michaelis-Menten functions (red curves). This allowed us to compare contrast gain (g) of amplitude versus contrast functions for K2.1 and K2.2. The difference in contrast gain was aligned with the hypothesis that K2.1 is dominated by M-pathway contribution while K2.2 is dominated by P-pathway contribution. However, the difference in saturation indicated that they are similar or even contrary to the above hypothesis. One possibility is that K2.1 has a mixed contribution of the M- and P-pathways. Error bars are SEM.

**Figure 4:**
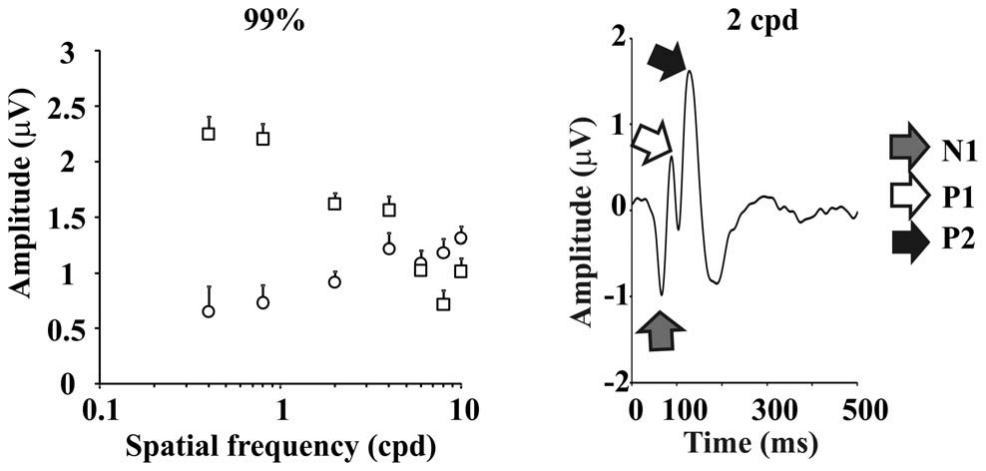
VECP components of the second order kernel first slice (K2.1), N1, P1 and P2 (right). Mean amplitude of the VECP components of the second order kernel first slice along the spatial frequency domain at high contrast stimulation (left). Circles and squares represent P1 and P2 components, respectively. They showed opposite spatial frequency tuning: P1 amplitude was high-pass tuned while P2 amplitude was low-pass tuned. Error bars in the left panel are SEM.

The contrast and spatial frequency influences in the VECP kernel mean RMS amplitude are shown in Figure 3. The K1 mean RMS amplitude was very small along the contrast domain at all spatial frequencies. The K2.1 mean RMS amplitude increased with contrast at all spatial frequencies and saturated at high contrasts. This behavior was more pronounced at low and intermediate spatial frequencies. On the other hand, the K2.2 mean RMS amplitude increased linearly with contrast at low and high spatial frequencies and showed some saturation at high contrast for intermediate spatial frequencies. We have fitted the contrast response mean values with power functions and Michaelis-Menten functions (Figure 3). K2.1 functions have larger z than K2.2 functions, especially at low spatial frequency (Figure 3). In addition, K2.1 is more sensitive than K2.2 to contrast as shown by its larger contrast gain (Figure 3). The results of contrast gain (g) estimate together with the spatial frequency sensitivity of each slice (Figure 4) made us to suggest that K2.1 is M-pathway dominated and K2.2 is P-pathway dominated. However, the results of saturation analysis were inconclusive and might even suggest the contrary. We have also fitted the amplitude versus contrast functions obtained from each subject with both power functions and Michaelis-Menten functions and submitted the results for z and g values to ANOVA one way analysis. In spite of similar trend in the individual results as observed in the mean results, the large variability across subjects resulted in non-significant differences (p = 0.15).

### Effects of the Stimulus Spatial Frequency and Contrast on the VECP First Slice of Second Order Kernel of Components

We observed that on the K2.1 there was an earlier negative component (N1) that appeared at all spatial frequencies with similar amplitude and two positive components (P1 and P2) that were dependent on the spatial frequency. The criteria used to distinguish P1 and P2 were mainly the waveform morphology across the spatial frequencies. The names of the components P1 and P2 were determined by their latency in 2 to 4 cpd at 99% contrast where both usually appeared together. The variability in the presence of the K2.1 components for the group of studied subjects is shown in Table 1.

At low spatial frequencies, the VECP was dominated by a positive component, P2, whose amplitude decreased as a function of spatial frequency. At intermediate spatial frequencies there was an earlier positive component, P1, whose amplitude increased as a function of the spatial frequency (Figure 4). Figure 4 shows P1 (circles) and P2 (squares) amplitudes as a function of the spatial frequency.

**Figure 5:**
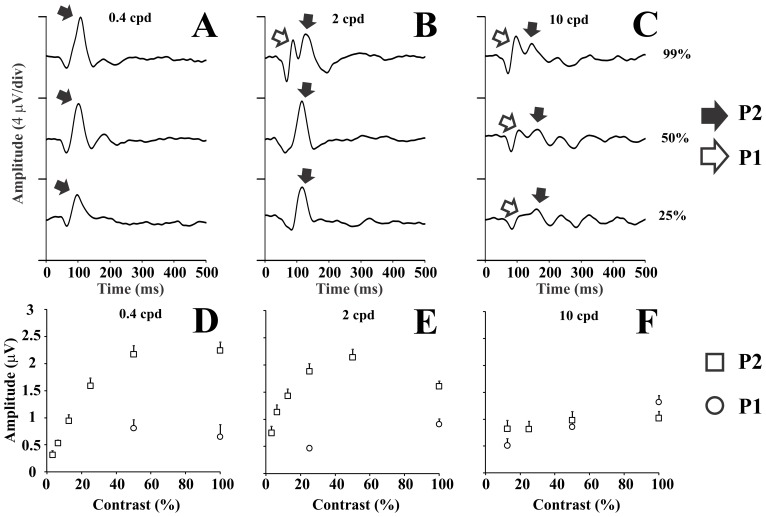
VECP components of the second order kernel first slice, P1 and P2, elicited by stimulus at different contrast levels. (A–C) VECP waveforms at three contrast levels. (D–F) Component amplitudes as a function of stimulus contrast. At low spatial frequency, P2 component dominated at all contrast levels. At intermediate spatial frequency, P1 and P2 components co-existed only at high contrast. When contrast was lowered, P1 component became very small while P2 component remained large. At high spatial frequency, both components were present at 25–99% contrast levels and, in addition, P1 is larger than P2 at the highest contrast level. Error bars in the lower panels are SEM.

Figure 5 (top row) shows K2.1 components waveforms for three spatial frequencies at three contrasts. Figure 5 (bottom row) shows the amplitudes of the K2.1 components as a function of contrast for three different spatial frequencies. At low spatial frequencies, P2 was present in the majority of contrast levels that were tested, decreasing in amplitude as the contrast was lowered, while P1 was measurable only at high contrasts and in some subjects. At intermediate spatial frequencies, P2 was present at all contrast levels and its amplitude decreased as contrast was lowered, while P1 was present mainly at high contrasts. At high spatial frequencies, P1 and P2 were present at high-to-intermediate contrast levels and their amplitude decreased linearly as a function of the contrast. At low and intermediate spatial frequencies, P2 amplitude as a function of the contrast saturated at high contrast.

### Effects of the Stimulus Spatial Frequency and Size on the VECP Components of the Second Order Kernel First Slice

**Figure 6:**
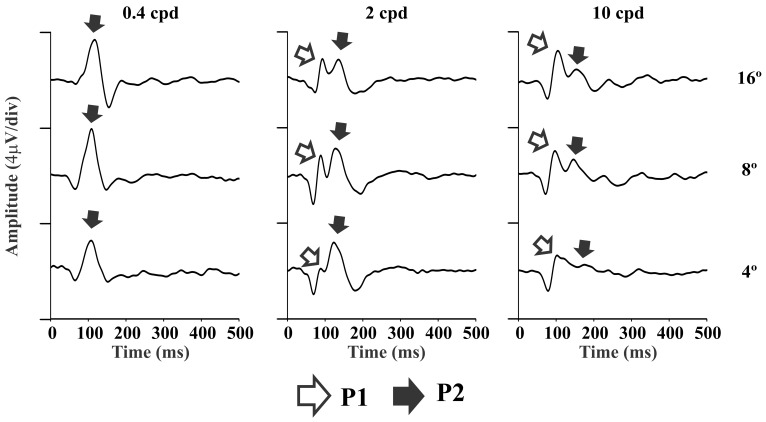
Influence of the stimulus size in the VECP waveforms of the second order kernel first slice (K2.1). At low spatial frequencies, the waveforms were similar for all stimulus sizes and the P2 component dominated the waveforms (left). At intermediate spatial frequencies, the P1 component was small or absent for small stimuli (4°) but was present for large stimuli (8° and 16°), while P2 amplitude was similar across all stimulus sizes (center). At high spatial frequencies, the two components largely overlap for small stimuli, but remained separated for large stimuli (left).

We also tested the role of stimulus size on the amplitude of the K2.1 components. We used stimuli with 4°, 8°, and 16° of visual angle at three spatial frequencies and high contrast. Figure 6 shows the mean waveform of the K2.1 elicited by different stimulus size. It was found that at low spatial frequencies, the VECP waveform was similar for the three tested stimulus sizes and that the P2 component dominated the waveform. At intermediate spatial frequencies the P1 component was present and its amplitude was stimulus size dependent: the P1 amplitude was small for small stimulus size, increasing when stimulus size was increased. The P2 component dominated the waveform at small stimulus size (4°) and it had similar amplitude across the range of stimulus sizes studied. At high spatial frequencies the separation between P1 and P2 components was difficult for small stimulus size (4°), but became less difficult for large stimulus size (8° and 16°).

### Principal Component Analysis

**Figure 7:**
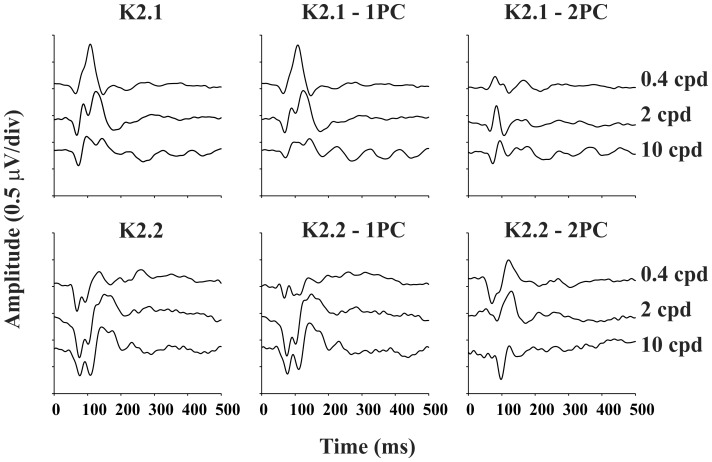
Mean VECP kernel waveforms obtained from 9 subjects at three spatial frequencies and 99% contrast. Left column: second order kernel first and second slices (K2.1 and K2.2, respectively). Center column: first principal component waveforms extracted from K2.1 and K2.2. Right column: second principal component waveforms extracted from K2.1 and K2.2.

We found two principal components in K2.1 and K2.2 slices. The variance explained by K2.1 principal components was about 49±5% for the first principal component and 20±3% for the second principal component, while the variance explained by K2.2 principal components was about 44±3% for the first principal component and 22±2% for the second principal component. The waveforms of K2.1 and of the first and second principal components extracted from the K2.1 are shown in the Figure 7 (top row). The first principal component waveform is very similar to the original K2.1. Both have the N1, P1, and P2 components. The second principal component waveform is similar, but smaller in amplitude than the K2.1 waveform. The K2.2. first and second principal components are dominated by a negative waveform.

**Figure 8:**
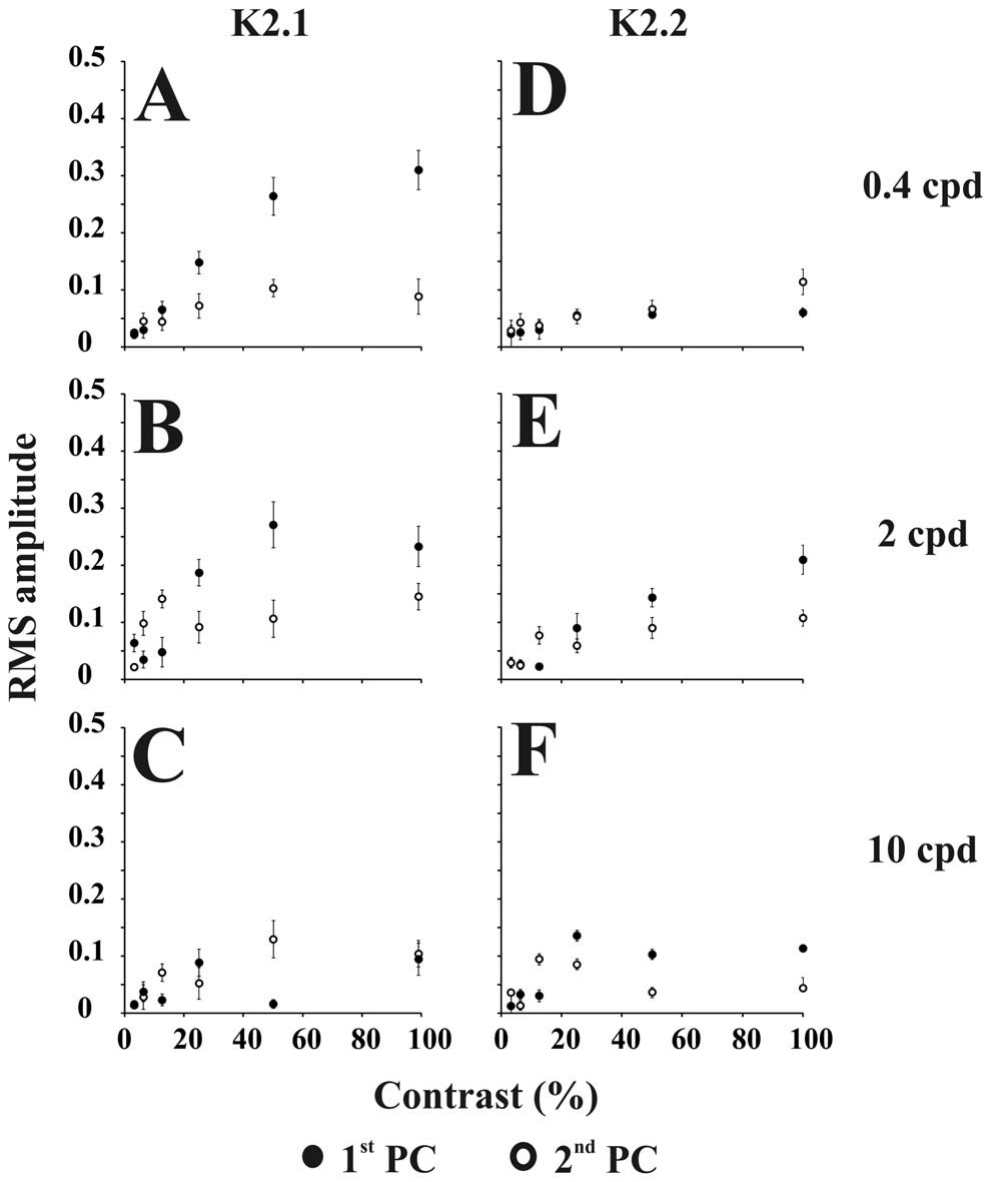
Contrast response functions for K2.1 first (filled circles) and second (empty circles) principal components at three spatial frequencies (0.4 cpd, 2 cpd, and 10 cpd) (A–C), and for K2.2 first (filled circles) and second (empty circles) principal components at the same spatial frequencies (D–F). K2.1 first principal component is more sensitive to contrast than K2.1 second principal component and K2.2 first and second principal components. We fitted power functions to the mean values (not shown for clarity) and observed that the largest difference between K2.1 first and second principal components were seen at 0.4 cpd (z = 0.84 and 0.37, respectively) and 2 cpd (z = 0.57 and 0.38, respectively) and between K2.2. first and second principal components were seen at 2 cpd (z = 0.57 and 0.37, respectively). We fitted Michaelis-Menten functions to the mean values (not shown for clarity) and observed that the largest difference between K2.1 first and second principal components were seen at 0.4 cpd (g = 1.06 and 0.31, respectively). The difference in contrast gain are suggestive that K2.1 first principal component is dominated by M-pathway response, whereas the other components are dominated by the response of a less contrast sensitive pathway such as the P-pathway. Error bars are SEM.

Figure 8 shows the mean contrast response functions for the first and second principal components extracted from K2.1 and K2.2. We fitted power functions to the mean values and the largest difference was found between K2.1 first and second principal components at low and intermediate spatial frequencies (0.4 and 2 cpd, respectively; first principal component z larger than second principal component). We have also fitted Michaelis-Menten functions to the data and estimate the contrast gain. Contrast gain was larger for the first principal component in comparison with the second principal component. The difference in contrast gain are suggestive that K2.1 first principal component is dominated by M-pathway response, whereas the other components are dominated by the response of a less contrast sensitive pathway such as the P-pathway. However, the results of saturation analysis were inconclusive and might even suggest the contrary.

**Figure 9:**
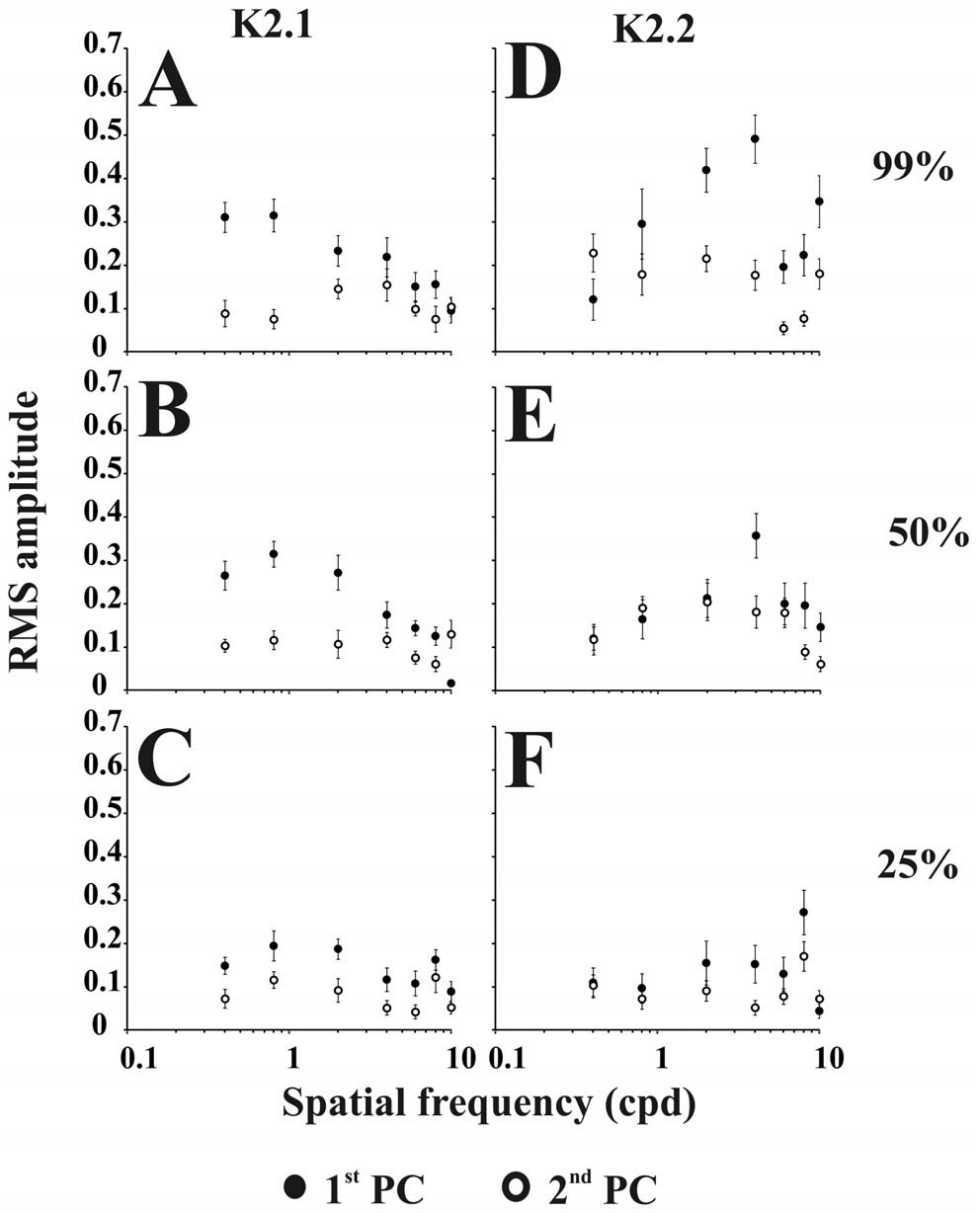
Spatial frequency response functions for K2.1 first and second principal components at three contrast levels (99%, 50%, and 25%) (A–C), and for K2.2 first and second principal components at the same contrast levels (D–F). K2.1 first principal component amplitude generally decreases as spatial frequency increases whereas K2.2 second principal component amplitude and amplitudes of K2.2 first and second components generally increases when spatial frequency is increased. These results also suggest that the K2.1 first principal component is dominated by M-pathway response whereas the other components are dominated by P-pathway response. Error bars are SEM.

Figure 9 shows the RMS amplitude of the first and second principal component extracted from K2.1 and K2.2 at three contrast and spatial frequencies. At 99% contrast, K2.1 first principal component, K2.1 second principal component, and K2.2 second principal component were tuned to low spatial frequency, while K2.2. first principal component was band-passed tuned. As the contrast decreased, they kept almost the same mean level across the spatial frequencies.

## Discussion

A hot topic in basic visual neuroscience with potential large applications in ophthalmology and neurology is to devise methods to separate the contribution to vision of the parallel pathways that connect the retina, the subcortical visual centers, and the primary visual cortex [10]. Among these streams of visual information, the M and P pathways are the best studied and deal with different aspects of visual perception [10]. Previously, it was suggested that the use of m-sequences to evoke visual cortical potential has the potential to separate the activity of different neural sources of visual responses in the time domain [11], [18], [24]. In the present work we described the influence of spatial parameters in the VECP waveforms for different kernels. The waveform analysis showed evidence for two contrast processing mechanisms working at different spatial frequency ranges. A principal component analysis also showed two mechanisms that contribute to the pseudo-random VECP.

We consider as the main contribution of the present work, the study of the influence of the spatial frequency and contrast in the cross-correlated cortical response elicited by pseudo-random stimulation. Our findings suggest the existence of at least two mechanisms to detect the spatial contrast, as shown for temporal contrast [11]. We propose that the contribution of M- and P-pathway could explain most of our results, but not all of them.

### Influence of the Stimulus Spatial Frequency and Stimulus Contrast on the Conventional VECP

Our findings showed that the waveform of the K2.1 was dependent of the spatial frequency and contrast (Figures 1–3). We have observed the appearance of two positive components in K2.1 and studied their behavior when the spatial frequency, contrast, and stimulus size were changed (Figures 4–6).

Previous results have shown two pathways contribution to the conventional VECP [1]–[6]. We reported that the VECP can be differentially generated by visual stimuli comprising at least three different ranges of spatial frequencies: at low spatial frequencies, 0.4–0.8 cpd, VECP generation was M-pathway dominated; at intermediate spatial frequencies, 2–4 cpd, VECP generation received contribution of both the M- and P-pathway; and at high spatial frequencies, 8–10 cpd, the P-pathway played a major contribution to VECP waveforms [4], [6]. Our findings indicated that the M-pathway activity dominates the VECP generation at low and intermediate spatial frequencies and that the P-pathway activity progressively increases its influence on the VECP when spatial frequency is raised. Similar conclusions were suggested by other authors that studied VECP using temporal changes of stimulus luminance [1]–[3].

Several previous studies have tried to characterize how the stimulation with different ranges of spatial frequencies contributes to the generation of visual evoked potentials, describing changes in the VECP waveforms as a function of spatial frequency [4], [14], [30]–[37]. These works used conventional periodical visual stimulation in the time domain with grating patterns and averaged evoked potential recording. They substantially differ from our work once we used binary m-sequence pseudorandom visual stimulation and cross-correlation analysis of the VECP waveform.

Campbell & Maffei [14] found the spatial frequency dependence of VECP amplitude versus contrast. For functions elicited by gratings with spatial frequency above or below 3 cpd, they found double slope functions or linear functions, respectively. They suggested that retinal mechanisms located in the fovea and parafovea were responsible to generate these different functions.

Parker & Salzen [30] described early (N1–P1) and late (N2–P2) VECP waves bearing different relationships with the stimulus spatial frequency content: N1–P1 amplitude was consistently greatest at low spatial frequencies while N2–P2 showed consistent attenuation at low spatial frequencies. Jones & Keck [31] described a VECP negative-positive component complex at spatial frequencies below 3 cpd, while at above 3 cpd the negative and positive components were better separated and the N1 component was then followed by the positive component. They suggested that the significance of the negative-positive component complex represents the response of a transient system due to its appearance at low spatial frequency and its saturation at low contrasts.

Plant et al. [33] described that in some subjects of their sample (7/13 subjects) the VECP waveforms had two positive peaks. The stimulus conditions that favored the appearance of the two peaks were low spatial frequency and large visual field stimulus. They reported that the peak separation never persisted above 2 cpd. Reed et al. [34], using grating onset presentation at spatial frequencies ranging from 0.5 to 8 cpd, found two negative-positive complexes that were amplitude tuned at high spatial frequencies (earlier complex) and low spatial frequencies (late complex). Jones & Keck [31], Plant et al. [33], and Souza et al. [4] found in some subjects two positive peaks in the P100 component at intermediate spatial frequencies.

Strasburger et al. [38] reported evidence from factor analysis of dichotomous mechanisms in the VECP amplitude as function of spatial frequency. One mechanism was specialized at low spatial frequency and the other at high spatial frequency. The signals from both mechanisms partially cancelled at intermediate spatial frequencies (about 3–4 cpd) [38], [39].

### Influence of the Stimulus Contrast and Spatial Frequency on the Pseudo – Random VECP Waveforms

Previously, Klistorner et al. [11] have found evidences of two contrast detection mechanisms in the pseudo random VECP waveforms elicited by temporal changes in the stimulus luminance. They found for the first order kernel the interplay of both mechanisms, and differential contribution of these mechanisms to the generation of the K2.1 and K2.2. They used the VECP amplitude as a function of the stimulus contrast to suggest that the M-pathway contributed to the K2.1 due to the saturation of the amplitude at high contrast, and that the P-pathway contributed to the K2.2 due to the linear amplitude versus contrast functions of that kernel. Our results seem to be in according with those obtained by Klistorner and colleagues, despite the differing frame rate causing their slices to be separated by 15 ms, rather than the 13.3 ms here. To exploit further this issue, we performed a detailed analysis of the variation of K2.1 and K2.2 amplitude with contrast by fitting the data point with power functions and Michaelis-Menten functions (Figure 3). This allowed us to compare both contrast gain and saturation of amplitude versus contrast functions for K2.1 and K2.2. The difference in contrast gain was aligned with the hypothesis that K2.1 is dominated by M-pathway contribution while K2.2 is dominated by P-pathway contribution. However, the difference in saturation indicated that they are similar or even contrary to the above hypothesis. One possibility is that K2.1 has a mixed contribution of the M- and P-pathways.

The absence of responses in the K1 in the present investigation is possibly due to the symmetry of the cortical responses for the pattern reversal stimulation. Klistorner et al. [11] got large first order components because the luminance of their hexagons changed in time, in our case the gratings are on average equi-luminant and so no first order kernel is expected because the linear responses to the dark and light components cancelled. Baseler et al [20] used both the luminance modulation method and the contrast reversal. They compared the types of first order kernels obtained in both conditions.

We have also found that the spatial frequency tuning is different for the K2.1 and K2.2 (Figure 2). TheK2.1 had low-pass tuning in the spatial frequency domain (an M-pathway property), while the K2.2 was band-pass tuned in the same domain (a P-pathway property). As the stimulation used by Klistorner and co-workers had no spatial contrast, the better comparison of their results is with our results for low spatial frequencies. There is a good similarity between the K2.1 and K2.2 waveforms and amplitude versus contrast functions from both studies (Waveforms: Figure 1, this work; Figure 5A–B, Klistorner et al., [11]; Amplitude versus contrast function: Figure 3, this work; Figure 5C–D, Klistorner et al., [11]). We understood that the present work extended the findings of Klistorner et al. [11] for other spatial frequencies where the interplay of M- and P-pathway could have other weights.

Momose [24] studied the pseudo random VECP at different spatial frequencies (between 0.5–4 cpd) and looked for correlations with steady-state VECP elicited by low, intermediate, and high temporal frequencies. The rationale was that steady state VECP elicited by low and high temporal frequencies was dominated by the P-pathway and M-pathway contribution, respectively. She found that latency of the 150 ms peaks of the second order kernel first and second slices were correlated with steady-state VECP elicited by high temporal frequencies, while the amplitudes of the second and third slices of the second order kernel were correlated with steady state VECP elicited by intermediate temporal frequencies (4–16 Hz). The latencies of the second order kernel third slice were correlated to the steady state VECP evoked by low temporal frequencies. She suggested that the K2.1 reflected the M contribution, the K2.2 had a joint contribution of the M- and P-pathways, and the second order kernel third slice was dominated by the P-pathway contribution. Momose’s frame rate was 100/s so her K2.3 corresponds closely with our K2.2. Our RMS amplitude results agreed with those from Momose [24] that the different slices would reflect the M- and P-pathway differentially, but the analysis of the VECP components of the K2.1 also showed some P-pathway contribution.

### Influence of the Stimulus Contrast on the Pseudo – random VECP Components

Baseler & Sutter [18] used dartboard patterns to study the multifocal VECP across the contrast domain in different visual field eccentricities (0.2 to 6.4 degrees). They decomposed the responses into two additive components, an early C1 component attributed to the M-pathway activity and a late C2 component associated with the P-pathway activity. C1 and C2 contrast-response functions were compatible with the visual responses of retinal and geniculate M and P neurons as determined by several research groups [12], [13], reviewed by Silveira et al. [10]. The M component saturated at or above 13% contrast and it was absent or very small in the waveforms elicited by equiluminant red-green stimulation. The P component increased linearly from 4% to 53% contrast, saturated at high contrast, and had high amplitude for equiluminant red-green stimulation.

In this work, using sine wave gratings presented in temporal pseudorandom sequences, we also found VECP with double-peaked waveforms mainly at intermediate spatial frequencies (Figures 4–6). The early component, P1, occurred at high and intermediate contrasts (25–99%) as well as intermediate and high spatial frequencies (2–10 cpd) (Figures 4–5). The late component, P2, occurred at most contrasts and spatial frequencies, including the lowest tested contrasts at low spatial frequencies, becoming very small or disappearing only for stimuli combining high spatial frequencies (4–10 cpd) and low contrasts (12.5% or less) (Figures 4–5).

One way to look at our and Baseler & Sutter [18] results together is to compare contrast response functions obtained with sine wave grating stimulus of low spatial frequency (this work) with those obtained with stimulation by the dartboard peripheral sector large checks, and similarly to compare contrast response functions obtained from high spatial frequency stimulation (present work) with those obtained with stimulation by dartboard central sector small checks. P1 and P2 contrast response functions were similar to C1 and C2 contrast response functions, respectively (low spatial frequency: Figure 5, this work, and Figure 8a 6.4 deg of eccentricity, Baseler & Sutter, [18]; high spatial frequency: Figure 5, this work, and Figure 8a 0.2 deg of eccentricity, Baseler & Sutter, [18]). P2 and C2 contrast response functions had similar shape. At higher eccentricities, C2 had RMS amplitude saturation at the high contrasts as well as P2 at low spatial frequencies. Although the similarity of the contrast response functions between these components, our suggestion based in the measurement of the contrast gain is that P2 is M-pathway signature, while Baseler & Sutter [18] considered it as reflection of P-pathway. Kaplan & Shapley [13] showed that M ganglion cells saturated at high contrast, while P ganglion cells increased their activity almost linearly across the contrast. In addition, they also showed that M ganglion cells had high contrast gain while P ganglion cells had low contrast gain [13]. Baseler & Sutter [18] based their suggestion in the P and M cells distribution across the retina and the presence of C2 at isoluminant red and green stimuli. Baseler & Sutter [18] found that C1 also increased linearly the amplitude as a function of the contrast. Our proposal is not in agreement with that made by Baseler & Sutter who considered C1 as reflecting M-pathway activity based in the same arguments about C2 origins. Abdullah et al. [40] showed contrast response functions of the multifocal steady-state VECP that had a dip around 7% of contrast that was more prominent in the central field. They argued that at about 7% of contrast M and P cells could be responding in different phases and it led a partial cancellation of both signal.

Studies in the thalamus and visual cortex of non-human primates have determined that M (magnocellular) cells have shorter mean onset latencies than P (parvocellular) and K (koniocellular) cells [41]–[46]. The M cell advantage over the other cell classes varies between 13 ms to 20 ms [43], [44], [46]–[49]. On the contrary, some other studies have not found any difference between the visual pathway velocity conduction pathways. Spear et al. [50] were able to make quantitative comparisons between M and P neurons using larger samples than those that have been studied previously. They found that M neurons had significantly higher maximal response rates and signal-to-noise ratios than P neurons but response latencies to visual stimulation were similar for neurons in the two types of lateral geniculate layers. Maunsell et al. [42] suggested that the P-pathway could reduce the M-pathway advantage in the timing of the inputs to visual cortex because the number of P cells converging to the visual cortex entrance layers is one order of magnitude larger than those of the M cells [51], [52] generating more input in V1 than the M cells do. So, the signal-to-noise ratio of the P-pathway would be larger, turning its visual response earlier detectable than that of the M-pathway [42]. Ellemberg et al. [37] showed that an earlier VECP component, N1, was tuned to high spatial frequencies at high contrasts reflecting P-pathway activity, while a later component, P1, would represent M-pathway activity. Ellemberg et al. [37] and Previc [53] suggested that although P1 appeared later than N1, the P1 onset could be prior than N1 but it occurred hidden inside N1 onset.

### Influence of the Stimulus Size on the Pseudo–random VECP Components

Allito et al. [54] studied the responses of the lateral geniculate nucleus neurons in alert macaque. They found that in eccentricities below 3°–5°, the most responsive cells were almost exclusively M cells. P cells fired mainly after 3° of eccentricity. We tested the hypothesis of Allito and co-workers to the generation of the P1 and P2 components of the second order kernel first slice. We hypothesized that small stimulus size would elicit M-dominated cortical responses, and larger stimulus sizes would evoke the activation of M- and P-pathways. We stimulated using 4°, 8° and 16° of stimulus size. The VECP waveforms for different stimulus sizes were very similar at low spatial frequencies and were dominated by the P2 component (M-pathway activation) (Figure 6). However, the VECP waveforms for different stimuli sizes were different at other spatial frequencies, mainly at intermediate spatial frequencies (2–4 cpd). The P2 component was similar for different stimulus size, while P1 component was almost absent for stimulation using 4° of size, but it was present for stimuli sizes of 8° and 16°. We considered reasonable to interpret these findings as differential contribution of M and P to the VECP generation.

Thus, we suggested that the P2 component, in spite of having longer implicit time than P1, was associated with the activation of the M-pathway once its response to visual stimulation had the features associated to M cells: very sensitive to luminance contrast and more responsive to low and intermediate spatial frequencies. On the other hand, the P1 component, in spite of having shorter implicit time than P2, was associated with the activation of the P-pathway once its response to visual stimulation had features associated to P cells: relatively insensitive to luminance contrast and more responsive to intermediate and high spatial frequencies.

### Principal Component Analysis and Pseudo-random VECP

We found that K2.1 waveforms were generated by the interaction of two mechanisms with different contrast and spatial frequency selectivity, while K2.2 waveforms resulted from the intercourse between two mechanisms with similar response properties (Figures 7–9). The first principal component extracted from K2.1 was dominated by a positive component and it had features of M-pathway activity: high contrast gain at low spatial frequency (Figure 8) and low spatial frequency tuning (Figure 9). The second principal component extracted from K2.1 and both first and second principal component were dominated by a negative component and their analysis were associated with P-pathway activity: linear amplitude growth as a function of contrast and band-pass tuning in the spatial frequency domain. Other studies showed that negative components were associated to the P-pathway activity [7], [11], [37], [55].

### Conclusion

The results of this work reinforce previous suggestion of the separation (low and high spatial frequencies) and overlap (intermediate spatial frequencies) of two visual pathways in the cortical activity as measured with VECP recording [4].
